# The novel coronavirus (COVID-19) infection in Hangzhou: An experience to share

**DOI:** 10.1017/ice.2020.62

**Published:** 2020-03-05

**Authors:** MengYuan Diao, Sheng Zhang, Dechang Chen, Wei Hu

**Affiliations:** 1 Department of Critical Care Medicine, Affiliated Hangzhou First People’s Hospital, Zhejiang University School of Medicine, Hangzhou, Zhejiang, China; 2 Department of Critical Care Medicine, Ruijin Hospital, Shanghai Jiao Tong University School of Medicine, Shanghai, China

**Fig. 1.**
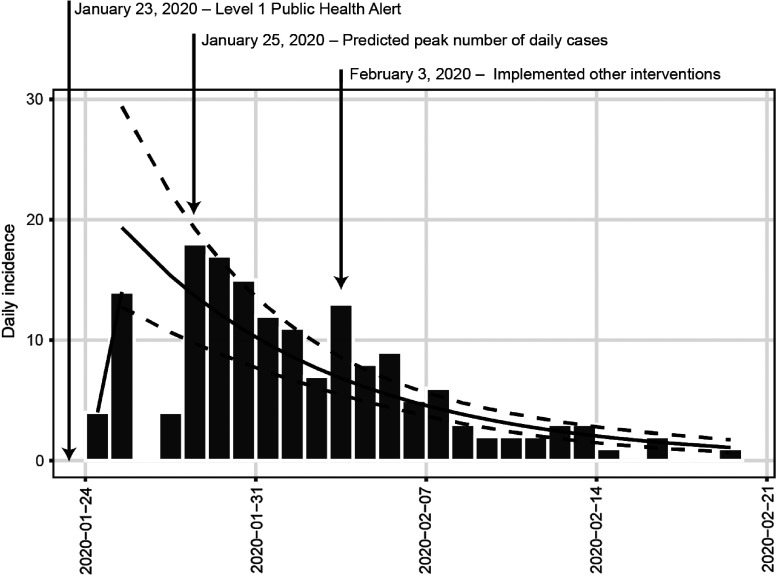
The impact of a public emergency health alert on the daily incidence of COVID-19 infection in Hangzhou. The fitted trajectory shows the probable daily incidence with 95% confidence interval derived from existing data using a log-incidence over time regression model. The split point is the optimal date to split the epicurve into two phases, which best fits the model. Other interventions include restricted movement outside the home, noncontact delivery, online work and teaching, etc.

*To the Editor*—Hangzhou, the capital of Zhejiang province in China, was confronted with the pandemic of a novel coronavirus (COVID-19) that originated in Wuhan, Hubei province.^1^ According to the Health Commission of Zhejiang Province,^2^ 6 cases were first reported on January 19, 2020, and the cumulative cases reached 169 as of February 20, 2020. The situation in Hangzhou was once rather severe—it was the top-ranking city with respect to the number of confirmed cases in Zhejiang province at the beginning of the epidemic. Since the Hangzhou government took rigorous measures to contain the epidemic, positive trends have been observed. The daily number of newly confirmed cases has sharply decreased within the last week, and only 1 case was confirmed from February 17 to 20. Similarly, Hangzhou reported no deaths in its administrative region. We used a regression of log-incidence over time model^3^ to provide a fitted trajectory for the actual daily incidence to verify the control effect. As shown in Figure 1, the optimal splitting point, defined as the peak number of daily new cases simulated by the model, occurred on January 25. This peak occurred about a week after launching the highest level of emergency public health alert and response in Hangzhou, which indicates that the prevention and control measures may have been effective.

Overall, 6 major measures were taken to control and prevent the spread of COVID-19 in Hangzhou. First, aware of the seriousness of the situation, on January 23, 2020, the Zhejiang province authorities launched a Level I Public Health Incident Alert, the highest level of emergency public health alert and response in the nation’s public health management system. As the top level of China’s public health alert system, this measure imposed the maximal limit on movement by people. Second, further action was taken on February 3, 2020, when most districts of Hangzhou announced that every community would be kept under closed management and that only 1 family member was allowed to leave the house to buy daily living supplies every 2 days. Third, “noncontact delivery,” a new delivery method, was adopted by many express delivery companies to reduce contagion risk. Fourth, to reduce the concentration of personnel to avoid the risk of cross infection, online working and network teaching were encouraged for workers and students, respectively. These measures were supported by mobile technology companies. Fifth, to meet the need to resume production and curb the transmission of the virus as far as possible, Hangzhou arranged chartered transportation to help numbers of migrants return to their work places. Lastly, in cooperation with Alipay, Hangzhou adopted the health quick-response (QR) code system on February 11, 2020, which were designated by green, yellow, or red. People who wanted to get into Hangzhou needed to submit their travel history and health information online in advance. Residents with a green code indicated they had a low current risk of being infected, while residents with yellow or red codes were quarantined for 7 or 14 days and were required to report their health condition daily to exclude infection before the code was changed to green. This health surveillance system has now been applied in most cities in Zhejiang province and will be promoted in other provinces.

Although the effect of prevention and control measures is evident, Hangzhou continues to face huge challenges owing to its large immigrant population. However, this city has already learned much from this epidemic, and we hope that some of our experiences will assist others in their regions.
